# Re-description of *Parasphaerosyllisindica* Monro, 1937 (Annelida, Syllidae), with the establishment of a new species from western Mexico

**DOI:** 10.3897/BDJ.12.e116082

**Published:** 2024-01-31

**Authors:** Gerardo Góngora-Garza, María Ana Tovar-Hernández, Jesús Angel de León-González

**Affiliations:** 1 Universidad Autónoma de Nuevo León, San Nicolás de los Garza, Nuevo León, Mexico Universidad Autónoma de Nuevo León San Nicolás de los Garza, Nuevo León Mexico

**Keywords:** Polychaeta, Syllinae, Gulf of California, *Parasphaerosyllismalimalii*, Tropical Eastern Pacific

## Abstract

**Background:**

*Parasphaerosyllis* Monro, 1937 is a syllid genus, currently composed of four species: *P.indica* Monro, 1937 from the Arabian Sea, *P.uschakovi* (Chlebovitsch, 1959) from the Kurile Islands, *P.ezoensis* Imajima & Hartman, 1964 from Japan and *P.malimalii* Capa, San Martín & López, 2001 from the Pacific coast of Panama. The distribution of *P.indica* is circum-tropical to temperate waters, but the presence of species complexes has been suggested. In order to clarify the distribution of *P.indica* in many areas of the world, a re-description, based on examination of the type material, is required as a first step to a better understanding of its diagnostic features.

**New information:**

*Parasphaerosyllisindica* is re-described, based on holotype examination, a new species is established from the Gulf of California and *Parasphaerosyllismalimalii* is reported for the first time since its description in 2001. *Parasphaerosyllisirregulata* sp. nov. is distinguished from its congeners by the following features: 1) Palps are free at their base; 2) Two types of dorsal cirri are present: spherical to bulbous and moniliform cirri; 3) Both types of cirri are distributed irregularly. A spherical/bulbous and moniliform cirrus may appear together within the same segment (asymmetrical segment) or only a spherical/bulbous cirrus may appear in several consecutive segments (not alternating as occurs in congeners); 4) The spherical/bulbous cirri may have distal knobs with 1–3 terminal articles; and 5) Bidentate falcigers with short, sub-triangular blades with a proximal tooth slightly larger that the distal one. A taxonomic key to species of *Parasphaerosyllis* species is included.

## Introduction

Syllidae Grube, 1950 (Grube 1850) is the most speciose annelid family, with 700 species according to San Martín and Aguado (2019) or nearly 1,000 species described worldwide according to Pamungkas et al. (2019). Regarding Mexico, Syllidae is the second most speciose family after Nereididae de Blainville, 1818 (de Blainville 1818), with 83 species reported in its territory (Tovar-Hernández et al. 2014). Despite the Mexican polychaete fauna having been widely studied over the last century (Salazar-Vallejo and de León-González 2021), there are few comprehensive taxonomic investigations that focus on syllids. Except for some of the Rioja's contributions (Rioja 1941, Rioja 1943, Rioja 1958, Rioja 1962), the vast majority of studies provides faunistic lists or ecological analysis (e.g. Bastida-Zavala (1993), Hernández-Alcántara et al. (2017)), whereas the inclusion of detailed morphological descriptions, illustrations of key features and analysis or discussions of interspecific variation are scarce (Góngora-Garza and de León-González 1993, Díaz-Castañeda and San Martín 2001, Ruiz-Ramírez and Harris 2008, Tovar-Hernández et al. 2008, Góngora-Garza et al. 2011, Ruiz-Ramírez 2011, Salcedo-Oropeza et al. 2011, Salcedo-Oropeza et al. 2012a, Salcedo-Oropeza et al. 2012b, Salcedo-Oropeza et al. 2016).

*Parasphaerosyllis* Monro, 1937 (Monro 1937) is a genus, characterised by the presence of dorsal cirri from the mid-body alternating between long, strongly annulate cirri and short, lemon-shaped cirri (San Martín et al. 2008, San Martín and Aguado 2019). However, it is remarkable that the term “lemon-shaped” or “bottle-shaped” *sensu* Hartmann-Schröder (1987) may be confused as discussed in the Remarks section. To date, *Parasphaerosyllis* includes four valid species: *P.indica* Monro, 1937, described from the Arabian Sea (coast of Oman) and reported worldwide in circum-tropical to temperate waters (Aguado et al. 2015); *P.uschakovi* (Chlebovitsch, 1959) (Chlebovitsch 1959) from the Kurile Islands (Sea of Okhotsk); *P.ezoensis* Imajima & Hartman, 1964 (Imajima and Hartman 1964), from off Shirikishinai (Hokkaido, Japan) and *P.malimalii* Capa, San Martín & López, 2001 (Capa et al. 2001) from Coiba (Pacific coast of Panama). In Mexico, Rioja (1958) and Salazar-Vallejo et al. (1987) reported *Parasphaerosyllisindica*, the former from Veracruz (Atlantic, Gulf of Mexico) and the latter from Concepción and Bacochibampo Bays (Pacific, Gulf of California). Unfortunately, specimens recorded in both contributions have been lost and, thus, re-examination is not possible to corroborate the presence of *P.indica* on the Mexican coasts.

## Materials and methods

### Fieldwork

Specimens of the new species described in the present study were collected in artificial reefs from La Paz Bay in the Gulf of California, Mexico. Methods are documented in de León-González and Balart (2016). An area of 0.20 × 0.20 m on a lateral wall of artificial-reef structures was sampled (0.04 m^2^; total sampled area by survey 0.4 m^2^). This area was fragmented with chisel and hammer and the fragments transferred to poly-ethylene bags *in situ*. Sorting and taxonomic analysis of formalin-fixed worm material was performed in the Laboratory of Biosystematics (UANL). Specimens of *Parasphaerosyllismalimalii* were collected by scraping rocks covered by coralline algae from the intertidal area of Nayarit and Jalisco (Central West of Mexico).

### Identification

Observations and body measurements were taken using an Olympus BX51 microscope with differential interference contrast (DIC). Photographs were taken with an attached Nikon D610 digital camera. Drawings were made with a camera lucida. A specimen was dehydrated in a series of progressive concentrations of hexamethyldisilazane (HMDS). Once air-dried, the specimen was mounted on aluminium stubs and gold-coated for observation in a JEOL JSM-6010Plus-LA scanning electron microscope at the Scanning Electron Microscopy Laboratory (LMEB), ECOSUR-Chetumal, Mexico.

## Data resources

Type material and additional materials were deposited in the Colección Poliquetológica, Universidad Autónoma de Nuevo León (UANL, NL-INV-002-05-09), El Colegio de la Frontera Sur (ECOSUR, QNR.IN.021.0497) and Colección Nacional de Anélidos Poliquetos de México, Instituto de Ciencias del Mar y Limnología, Universidad Nacional Autónoma de México (CNAP–ICML, UNAM, DFE.IN.061.0598). In addition, the holotype of *Parasphaerosyllisindica* was loaned for comparison from the British Museum of Natural History (BMNH) to the Zoological Museum Hamburg during a research stay by the authors (GGG and JALG).

## Taxon treatments

### Parasphaerosyllis indica

Monro, 1937

F4B9F78B-E2C4-525C-AFB9-D8418525E8CB

*Parasphaerosyllisindica* Monro, 1937: 273, text-fig. 8.— Fauvel 1939: 298 (Annam Sea, French Indochina); Fauvel 1950: 351 (Dakar, Senegal); Fauvel 1953: 162, fig. 80c–d (Arabian coast); Rioja 1958: 246–251, figs. 21–27 (Isla Verde, Gulf of Mexico); Fauvel and Rullier 1959: 514–515 (Gorea Island, Senegal); Hartmann-Schröder 1965: 115 (Maui, Hawaii); Hartmann-Schröder 1980: 49 (Exmouth, northwest Australia); Hartmann-Schröder 1987: 32 (Victoria, Australia); Hartmann-Schröder 1991: 27 (Queensland, Australia); Rullier 1964: 165 (Cameroon); Rullier 1972: 69 (New Caledonia); Westheide 1974: 64–67, figs. 27–29 (Galapagos); Cantone 1976: 230 (Somalia); San Martín 1991: 234 (Cuba); San Martín et al. 2008: 146, figs. 19E–F, 22A–E, 24A–B (Tasman Sea and Western Australia); Liñero-Arana and Díaz-Díaz 2011: 24, figs 4.8–4.10 (Venezuela); Aguado et al. 2015: 49, figs. 5F (Lizard Island, Australia); Cañete 2017: 1070–1071, figs. 1a–g (Easter island, Chile).

#### Materials

- **Type status:** Holotype. **Occurrence:** occurrenceID: 5F89365A-89A3-5353-9EBC-8135225D86BE; **Location:** higherGeographyID: Western Indian Ocean; higherGeography: Arabian Sea; country: Oman; verbatimDepth: 13.5 m; decimalLatitude: 19.3767; decimalLongitude: 57.8833; **Event:** eventDate: 02-11-1933-11-02; year: 1933; month: 11; day: 2; **Record Level:** language: English; institutionID: BMNH; collectionID: BMNH; datasetID: BMNH 1937.9.2.156; collectionCode: Invertebrates; datasetName: BMNH

#### Description

**Figure 1.**
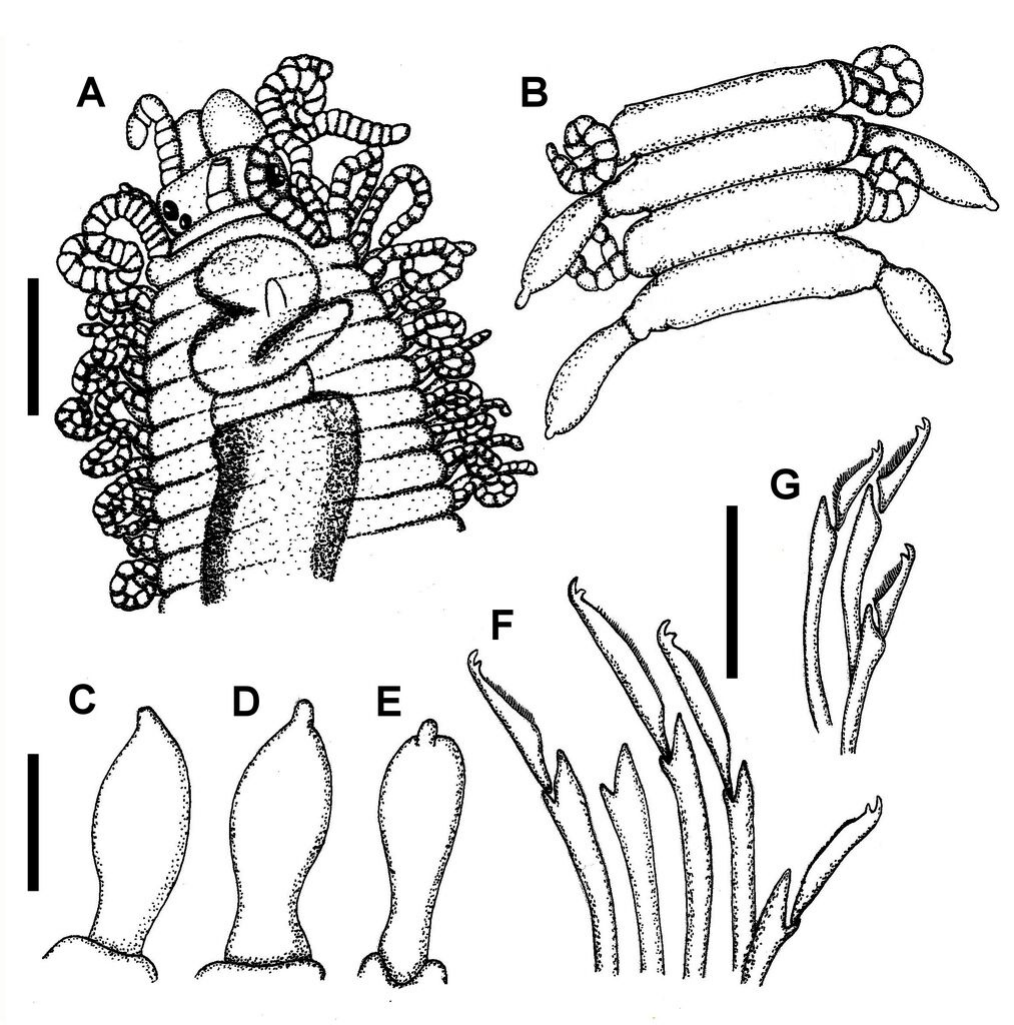
*Parasphaerosyllisindica* Monro, 1937 (holotype BMNH1937.9.2.156.). **A** Anterior end, dorsal view; **B** Middle anterior segments, dorsal view; **C** Bulbous dorsal cirrus, chaetiger 24; **D** Bulbous dorsal cirrus, chaetiger 40; **E** Bulbous dorsal cirrus, chaetiger 62; **F** Falcigers from chaetiger 10; **G** Falcigers from chaetiger 60. Scale bars: A, B = 0.2 mm; C, D = 0.1 mm; F, G = 20 µm.

Holotype incomplete posteriorly, 5 mm long, 0.4 mm wide on proventricle, 68 chaetigers. Body subcylindrical, ventrally flattened (segments 15 and 16 from anterior end remarkably wider than rest of body, perhaps due to manipulation caused by strong pressure on slide cover, because proventricle is broken and pharynx flattened). Body brown-yellowish, without pigmentation pattern. All cirriform annulated appendages deeply coiled (Fig. 1A). Prostomium pentagonal, wider than long. Four lensed eyes in trapezoidal arrangement, eyes from the anterior pair larger and more separate than posterior pair of eyes (Fig. 1A). Right anterior eye divided into two lobes, appearing to have two fused eyes. Eyespots absent. Palps stout, triangular, slightly directed towards the ventral side, similar in length to prostomium, only fused in a small part of base. Median and right antennae lost. The median antenna preserves the cirrophore and the first annulation only, which arises from posterior end of prostomium, between posterior pair of eyes (Fig. 1A). Left lateral antenna inserted on anterior margin of prostomium, longer than prostomium with 7 articles of different sizes in irregular arrangement (Fig. 1A). Peristomium shorter than subsequent segments, folding on posterior part of prostomium (Fig. 1A). Peristomium with two pairs of tentacular cirri: dorsal ones with 24 articles each, right ventral cirrus with 14 articles, left ventral cirrus incomplete, with 7 articles. First pair of dorsal cirri with 24 articles each. Dorsal cirri from chaetigers 2 to 17 with 20–24 articles, not alternating in length. Mid-body dorsal cirri with 20–22 articles and posterior cirri with 15–18 articles. The first bulbous cirrus appears on chaetiger 15 on left side, whereas on the right side appears on chaetiger 17. From chaetiger 17 backwards, dorsal cirri alternate regularly, one by one: an annulated cirri and a bulbous one. Bulbous dorsal cirri elongate, cirrophore not visible (Fig. 1B). Base of bulbous cirri thin, enlarged medium part and a small unarticulated knob at the end (Fig. 1B–E). Bulbous cirri with curved diagonal fibrous lines, being more evident on some cirri than others. Some annulated cirri broken (incomplete) or fully removed (only scars are evident). Parapodial lobes slightly wide on base, with one or two notorious acicular papillae. Ventral cirri digitiform, shorter or similar in length than parapodial lobe. Anterior parapodia with about 10 compound heterogomph chaetae (6 on chaetiger 1), number diminishes gradually through the body to 6 on posterior segments. Falcigers dorso-ventrally decreasing in length, bidentate blades with thin spines on its internal margin, shafts with small and fine distal spines. Falcigers from anterior chaetigers with blades longer and thinner than posterior ones (Fig. 1F–G). Size proportion between dorsal-most versus ventral-most blades: anterior one 2.0 to 2.1, posterior one 1.5 to 1.6. Simple acicular chaetae, dorsal or ventral, not observed. Anterior parapodia each with two slender aciculae: one straight, pointed and other distally curved, acute. Mid-body and posterior segments with one acicula per parapodium, larger and thicker than anterior ones, distally curved. Pharynx occupying the space of about seven segments, pharyngeal tooth located anteriorly, pointed, acute, yellowish. Proventricle broken, longer than pharynx, extending through 8–9 segments, with about 22 muscle cell rings, with distinct mid-dorsal line. From chaetiger 62 backwards, forming a male stolon (damaged).

#### Diagnosis

Palps fused at their bases. Peristomium shorter than following segments. Dorsal cirri from mid-body bulbous with a long distal end and terminal knob, alternating with long and articulate cirri in a regular pattern (one by one). Bidentate falcigers with long blades, proximal tooth shorter than distal one.

#### Distribution

*Parasphaerosyllisindica* was described from the coast of Oman to 13.5 m depth. It has been reported in many localities around the world. Unfortunately, many of these reports include only a brief mention or just the name within a list of species or tables of ecological analyses and compendia, making its status unverifiable: Hartman (1954) (Eniwetok Atoll); Reish (1968) Marshall Islands); Amoreux et al. (1978) (Gulf of Aqaba and Gulf of Suez); San Martín (1991) (Cuba); Núñez et al. (1992) and Pascual and Nuñez (1999) (Canary Islands); López and San Martín (1994) (Cape Verde Islands); Bastida-Zavala (1995) (Gulf of California, Mexico); Gómez et al. (1997) (Oaxaca, Mexico); Pleijel (2006) (New Caledonia) and Hutchings et al. (2014) (Australia). Aguado et al. (2015) already suggested that the presence of *P.indica* may represent a complex of species at least in Australia (Western Australia, Queensland, New South Wales and Victoria), but also it may be the same case in other disjunct localities.

On the other hand, several authors have reported *P.indica* through the decades, providing brief diagnoses/descriptions and illustrations that allow comparison with the re-description provided here (Table 1). The characters listed in Table 1 show great variability amongst all these records, demanding a detailed examination of voucher specimens. In addition, a study of ontogenetic variability is desirable, with special emphasis on the analysis of the following features that might be informative and consequently used in the recognition of species in *Parasphaerosyllis*:

- insertion of median and lateral antennae;
- extension of pharynx and proventricle and its size relationship;
- presence or absence of cirrophore in bulbous dorsal cirri;
- chaetiger where bulbous dorsal cirri appear;
- presence and number of terminal articulations in bulbous dorsal cirri;
- alternation pattern of moniliform/bulbous dorsal cirri (one by one or another pattern);
- presence of pseudosimple chaetae; dentition of falcigers;
- presence and distribution pattern of glands or pores in bulbous dorsal cirri (but this feature is only revealed using SEM).

#### Taxon discussion

**Figure 2.**
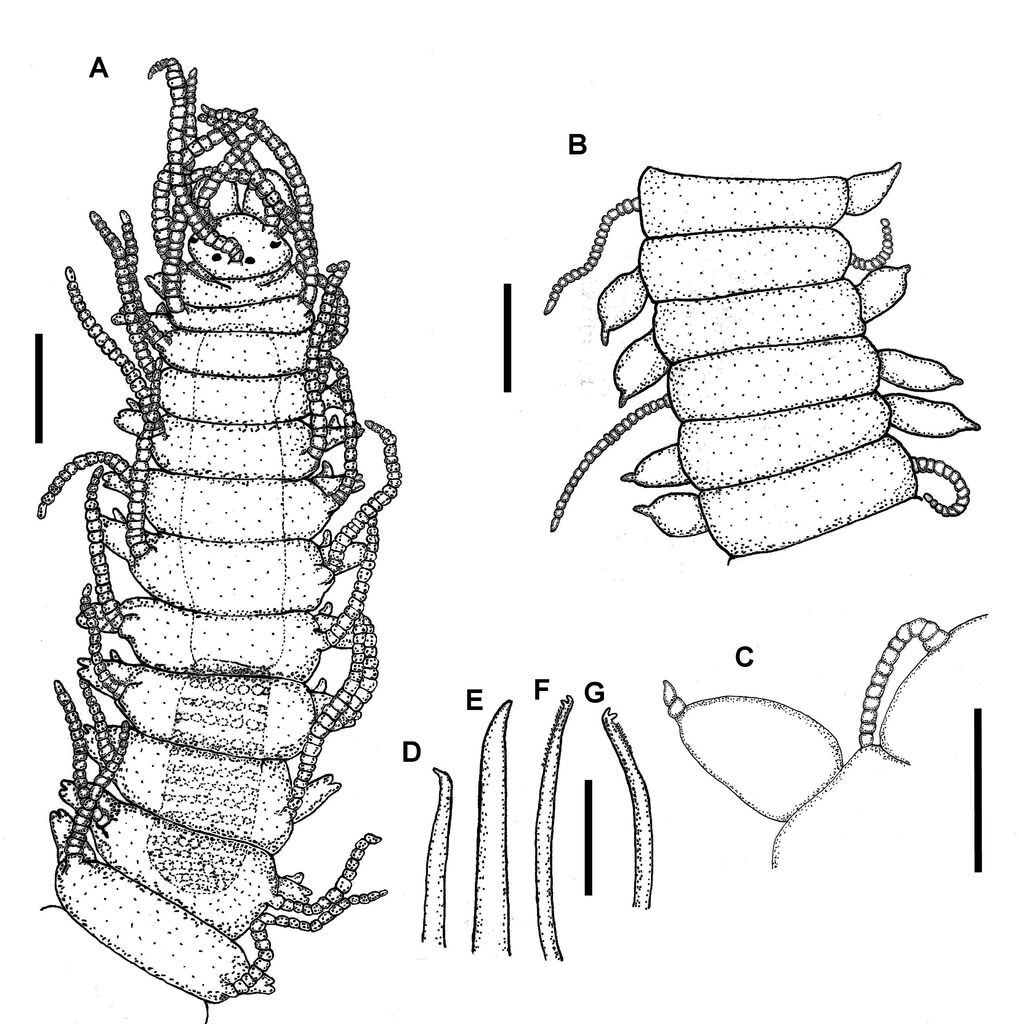
Line drawings of *Parasphaerosyllisirregulata* sp. nov. (holotype UANL 8158). **A** Anterior end, dorsal view; **B** Middle section of body, dorsal view; **C** Posterior bulbous and moniliform dorsal cirri, chaetiger 260; **D** Acicula, chaetiger 10; **E** Acicula, posterior chaetiger; **F** Ventral simple chaeta, chaetiger 302; **G** Dorsal simple chaeta, chaetiger 302. Scale bars: A, B = 0.5 mm; C = 0.25 mm; D–G = 20 µm.

Regarding the generic features of *Parasphaerosyllis*, the genus was established by Monro (1937) as having fused palps, except at the extreme tip in the type species (*P.indica*); and dorsal cirri from the mid-region including two types: moniliform (articulated or annulated) and bulbous, both being alternating (one by one). In recent generic diagnoses provided to *Parasphaerosyllis* by San Martín et al. (2008) and San Martín and Aguado (2019), it is stated that the genus has palps fused only basally and dorsal cirri from mid-body being short, unarticled, lemon-like alternated with articulate cirri. Additionally, the holotype of *P.indica* here examined has palps fused only basally, contrary to the original description by Monro (1937). Regarding the term dorsal cirri being “lemon-like”; in our opinion, this is subjective because it does not reflect the shape of these structures in all of the four species currently valid in the genus. Lemon-shaped can be spherical (as green lemon or lime) or ovoid (as yellow lemon) or pyriform and corrugated (as other lemon varieties). For instance, the dorsal cirri, originally described and illustrated by Monro (1937) (text fig. 8b), are bulbous with a terminal knob, which agree with the re-examination of the holotype (this study, Fig. 1B–E). In *P.malimalii*, dorsal cirri from the mid-region are ovoid or bulbous with a terminal knob (Capa et al. 2001, figs. 1B and D; this study, Fig. 6A), in *P.ezoensis*, these cirri are bulbous with knobs composed of two terminal articles (Imajima and Hartman 1964, plate 28, fig. b), in *P.uschakovi*, the cirri are bulbous with knobs composed of 2–3 terminal articles (Chlebovitsch 1959, fig. 1b and r) and, in the new species described below (*P.irregulata* sp. nov.), these cirri are spherical to bulbous and knobs have 1–3 terminal articles (Fig. 2B–C and Fig. 4C–E). Besides, the report of “*P.indica*” in Western Australia and Tasman Sea by San Martín et al. (2008) described the cirri as lemon-shaped, but it seem more like bulbous in the sense of this contribution.

Moreover, there is variation in the holotype of *Parasphaerosyllisindica* here examined versus the description by Monro (1937) (p. 274). He described the body as “slender and thread-like”, but we find that it is sub-cylindrical, widened in the first 15–16 segments to the posterior part of proventricle. The holotype is posteriorly incomplete, most of the stolon described by Monro having disappeared, perhaps due to over-handling of the specimen since other damage can be observed, such as the loss of the median and right lateral antennae.

According to the image provided by Monro (1937): fig. 8a, the insertion of the lateral antennae is located between the superior and the inferior pair of eyes, but in the holotype, lateral antennae are inserted on the distal margin of prostomium and the only visible antenna (left one) is incomplete, having only seven articles. The number of articles in the dorsal tentacular cirri varies; Monro illustrates tentacular cirri in his figure 8a with 20 articles on the left side and 21 on the right; but, the dorsal tentacular cirri in the holotype have 24 articles on both sides. Finally, Monro's figure 8b shows the bulbous cirri attached to the parapodium by a prominent cirrophore. When observing the holotype, we saw that parapodia do not have cirrophores and some cirri show different degrees of basal thinning (Fig. 1C–D).

### Parasphaerosyllis irregulata

Góngora-Garza, Tovar-Hernández & de León-González sp. nov.

51EF4C98-58F9-5DCB-9EFA-BF8298CB33B9

332180D6-F1CF-4F53-8366-D01A39D05138

#### Materials

- **Type status:** Holotype. **Occurrence:** catalogNumber: UANL 8158; occurrenceID: 32578C5D-D36F-5AC7-8996-310037A46514; **Taxon:** phylum: Annelida; class: Polychaeta; order: Phyllodocida; family: Syllidae; genus: Parasphaerosyllis; specificEpithet: *irregulata*; **Location:** higherGeographyID: Tropical Eastern Pacific; continent: America; waterBody: Gulf of California; country: Mexico; countryCode: MX; stateProvince: Baja California Sur; municipality: La Paz; locality: San Lorenzo Channel; verbatimDepth: 3 m; decimalLatitude: 24.3865; decimalLongitude: -110.315417; **Identification:** identifiedBy: Gerardo Góngora-Garza, Jesús Angel de León-González; **Event:** eventDate: 2015; year: 2015; month: May; day: 5; fieldNumber: 12; **Record Level:** institutionID: UANL, NL-INV-002-05-09; collectionID: UANL; institutionCode: UANL; collectionCode: UANL 8158
- **Type status:** Paratype. **Occurrence:** catalogNumber: UANL 8159; individualCount: 1; occurrenceID: CB4EEF8F-DBE8-50DA-9D83-75AFDCF623C2; **Taxon:** phylum: Annelida; class: Polychaeta; order: Phyllodocida; family: Syllidae; genus: Parasphaerosyllis; specificEpithet: *irregulata*; **Location:** higherGeographyID: Tropical Eastern Pacific; continent: America; waterBody: Gulf of California; country: Mexico; countryCode: MX; stateProvince: Baja California Sur; municipality: La Paz; locality: San Lorenzo Channel; verbatimDepth: 3 m; decimalLatitude: 24.3865; decimalLongitude: -110.315417; **Identification:** identifiedBy: Gerardo Góngora-Garza, Jesús Angel de León-González; **Event:** eventDate: 05/05/2015; year: 2015; month: May; day: 5; fieldNumber: 12; **Record Level:** institutionID: UANL, NL-INV-002-05-09; collectionID: UANL; institutionCode: UANL; collectionCode: UANL 8159
- **Type status:** Paratype. **Occurrence:** catalogNumber: ECOSUR 0000; individualCount: 1; occurrenceID: A0689572-82D7-51E0-86FC-2AD501C7A7F5; **Taxon:** phylum: Annelida; class: Polychaeta; order: Phyllodocida; family: Syllidae; genus: Parasphaerosyllis; specificEpithet: *irregulata*; **Location:** higherGeographyID: Tropical Eastern Pacific; continent: America; waterBody: Gulf of California; country: Mexico; countryCode: MX; stateProvince: Baja California Sur; municipality: La Paz; locality: San Lorenzo Channel; verbatimDepth: 3 m; decimalLatitude: 24.386917; decimalLongitude: -110.315111; **Identification:** identifiedBy: Gerardo Góngora-Garza, Jesús Angel de León-González; **Event:** eventDate: 05/05/2015; year: 2015; month: May; day: 5; fieldNumber: 2; **Record Level:** institutionID: ECOSUR QNR.IN.021.0497; collectionID: ECOSUR; institutionCode: ECOSUR; collectionCode: ECOSUR 0000
- **Type status:** Paratype. **Occurrence:** catalogNumber: CNAP–ICML 0000; individualCount: 1; occurrenceID: 369F3B1A-7D37-55AB-A9FB-577AB6B82D1C; **Taxon:** phylum: Annelida; class: Polychaeta; order: Phyllodocida; family: Syllidae; genus: Parasphaerosyllis; specificEpithet: *irregulata*; **Location:** higherGeographyID: Tropical Eastern Pacific; continent: America; waterBody: Gulf of California; country: Mexico; countryCode: MX; stateProvince: Baja California Sur; municipality: La Paz; locality: San Lorenzo Channel; verbatimDepth: 3 m; decimalLatitude: 24.386583; decimalLongitude: -110.315389; **Identification:** identifiedBy: Gerardo Góngora-Garza, Jesús Angel de León-González; **Event:** eventDate: 05/05/2015; year: 2015; month: May; day: 5; fieldNumber: 9; **Record Level:** institutionID: CNAP–ICML, UNAM, DFE.IN.061.0598; collectionID: CNAP–ICML; institutionCode: CNAP–ICML; collectionCode: CNAP–ICML 0000
- **Type status:** Paratype. **Occurrence:** catalogNumber: UANL 8160; occurrenceID: 807FE40C-F564-56E4-872E-F55DF8892634; **Taxon:** phylum: Annelida; class: Polychaeta; order: Phyllodocida; family: Syllidae; genus: Parasphaerosyllis; specificEpithet: *irregulata*; **Location:** higherGeographyID: Tropical Eastern Pacific; continent: America; waterBody: Gulf of California; country: Mexico; countryCode: MX; stateProvince: Baja California Sur; municipality: La Paz; locality: San Lorenzo Channel; verbatimDepth: 3 m; decimalLatitude: 24.386778; decimalLongitude: -110.315056; **Identification:** identifiedBy: Gerardo Góngora-Garza, Jesús Angel de León-González; **Event:** eventDate: 05/05/2015; year: 2015; month: May; day: 5; fieldNumber: 16; **Record Level:** institutionID: UANL, NL-INV-002-05-09; collectionID: UANL; institutionCode: UANL; collectionCode: UANL 816

#### Description

**Figure 3.**
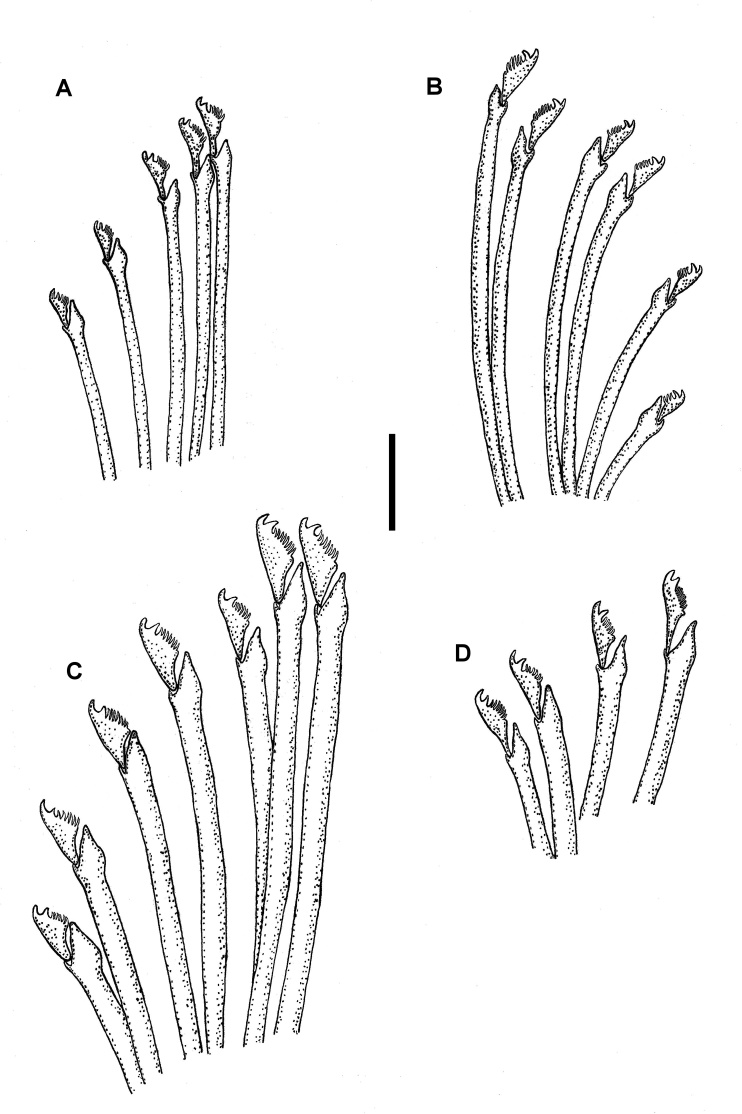
Line drawings of *Parasphaerosyllisirregulata* sp. nov. (holotype UANL 8158) **A** Falcigers from chaetiger 10; **B** Falcigers from chaetiger 40; **C** Falcigers from chaetiger 90; **D** Falcigers from chaetiger 250. Scale bar: A = 20 µm.

**Figure 4.**
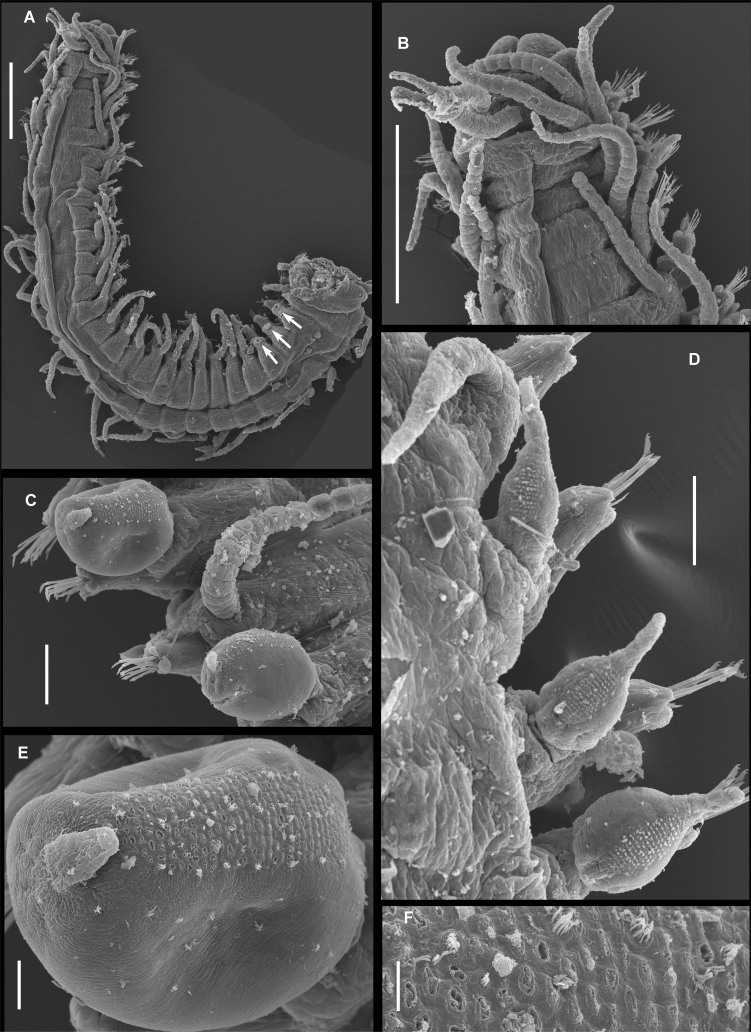
Scanning electronic micrographs of *Parasphaerosyllisirregulata* sp. nov. (paratype UANL 8160). **A** Anterior end, dorsal view, arrows point to insertion scars of dorsal cirri; **B** Prostomium and first anterior segments, dorsal view; **C** Alternated bulbous and moniliform dorsal cirri from mid-body region; **D** Posterior segments showing different development stages of bulbous dorsal cirri; **E** Bulbous dorsal cirrus showing dorsal band of several lines of pores; **F** Detail of ciliate pores on bulbous dorsal cirrus. Scale bars: A, B = 0.5 mm; C, D = 50 µm; F = 5 µm.

**Figure 5.**
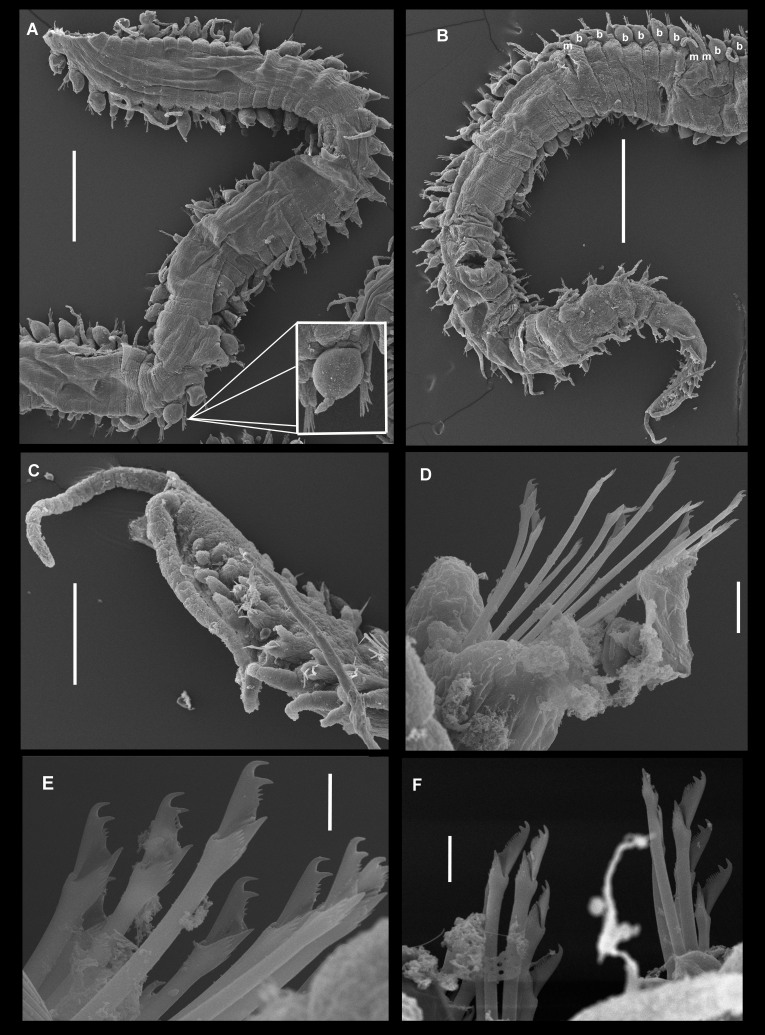
Scanning electronic micrographs micrographs of *Parasphaerosyllisirregulata* sp. nov. (paratype UANL 8160). **A** Middle section of the body, dorsal view, square showing an enlargement of a spherical cirrus with two terminal articles; **B** Posterior section of the body, dorsal view, where “b" means bulbous cirrus and “m” moliniform cirrus; **C** Terminal end, lateral view; **D** Anterior parapodium with falcigers; **E** Falcigers from middle body; **F** Falcigers from posterior parapodia. Scale bars: A, B = 0.5 mm; C = 0.1 mm; D, F = 10 µm, E = 5 µm.

**Figure 6.**
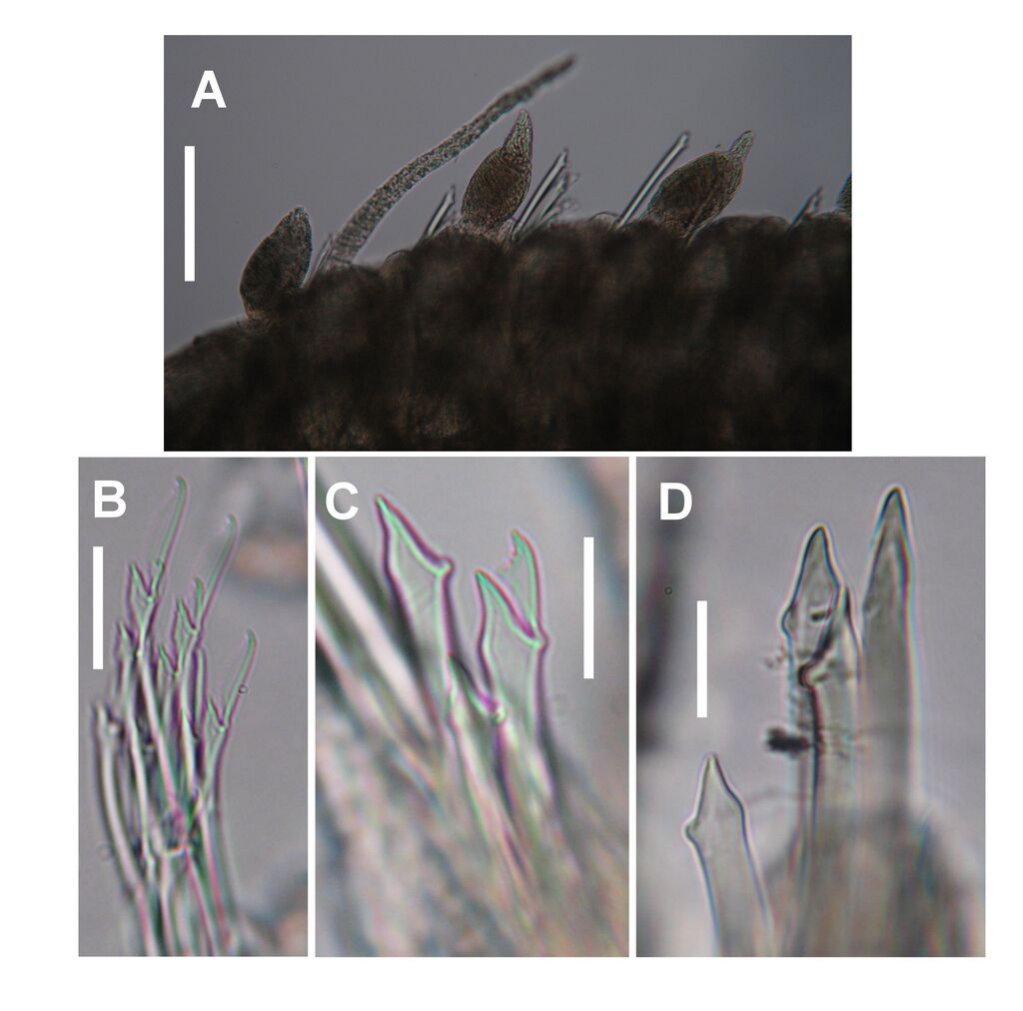
Digital photographs of *Parasphaerosyllismalimalii* Capa, San Martín and López, 2001 (UANL 8160). **A** Mid-body segments, showing bulbous and moniliform dorsal cirri; **B** Falcigers from chaetiger 10; **C** Falciger and pseudosimple chaeta from middle parapodium; **D** Pseudosimple chaetae from posterior parapodium. Scale bars: A = 50 µm; B–D = 20 µm.

Holotype complete, 35 mm long, 0.9 mm wide, 310 chaetigers. Body pale yellowish, without colour pattern, subcylindrical, ventrally flattened. Prostomium oval, wider than long. Two pairs of eyes in trapezoidal arrangement. Eyes of anterior pair longer than inferior pair, closer to external border of prostomium. Eyespots absent (Fig. 2A). Three annulate antennae. Median one with 26 articles, located in middle prostomium, between posterior eyes. Lateral antennae inserted in the anterior border of prostomium, with 16 articles each. All antennae longer than prostomium and palps. Palps subtriangular, slightly shorter than prostomium, free at their base (Fig. 2A). Peristomium as long as first chaetiger (Fig. 2A, Fig. 4A–B), with two pairs of tentacular cirri, dorsal ones with 24–25 moniliform articles, ventral ones with 13 articles each (Fig. 2A, Fig. 4A–B). Buccal hole with two large lateral edges and a central fissure formed by three irregular pleats. Dorsal cirri long, articulated. Articles near to cirrophore sub-rectangular, wider than long, following ones moniliform; distal article and, on occasions, subdistal ones longer than wide. First pair of dorsal cirri long, with 18 articles each. Cirri from second to sixth parapodia with 16–18 articles. After proventricle end, dorsal cirri alternating one by one (short and long), with 14–18 and 20–22 articles, respectively. In chaetiger 44 appear the first bulbous dorsal cirrus on right side only, with moniliform cirri on left side. From that region to the end of body, articulated cirri with 12–22 articles, with the exception of the last segments where it is growing and has few articles. Nearly spherical dorsal cirri in certain segments (Fig. 4C and E), while, in others, they are bulbous with distal part lengthened (Fig. 2B and Fig. 4D). The presence of spherical/bulbous cirri is irregular, not alternating one by one with moniliform cirri in all segments, but a series of up to 10 bulbous cirri can be present or 3 moniliform cirri in a row, although, sometimes, bulbous and moniliform cirri appear in the same segment forming asymmetric segments (Fig. 5A–B). Distal end of bulbous cirri with an unarticulated knob, smooth (Fig. 4C and E), although, in some cases, three or four terminal articles are present on posterior segments (Fig. 2C and Fig. 4D). Bulbous cirri with a dorsal band composed of several lines of pores (Fig. 4C–E, Fig. 5A–B and E–F), from which numerous groups of filaments or cilia are observed (Fig. 5E–F). Bulbous cirri present until before the last 20 chaetigers (Fig. 6B). Subconical parapodial lobe, truncate distally (Fig. 4D). Ventral cirri conical, distal end rounded, shorter than length of parapodial lobe. Anterior and median chaetigers with two aciculae per parapodia, one of these thicker than the other, both bent lightly in the distal end (Fig. 2D–E). Posterior parapodia with one acicula, similar in shape to the anterior ones. Anterior parapodia with 9–13 falcigers per fascicle, clearly bidentate with short blades, subequal teeth. Rate of size between dorsal-most versus ventral-most blades: 1.4 (Fig. 3A, 5D). Median parapodia with 10–13 bidentate falcigers. Blades of falcigers longer than anterior ones, secondary tooth slightly longer than apical one. Size proportion between dorsal-most versus ventral-most blades: 1.2 (Fig. 3B and Fig. 5E). Posterior parapodia with around 10 falcigers, similar of those of median parapodia, gradation dorso-ventral 1.5–1.6 (Fig. 3C). Pre-pygidial parapodia with 6–7 falcigers, larger than those of anterior parapodia, but similar in shape (Fig. 3D and Fig. 5F). Dorsal and ventral simple chaetae present on last eight chaetigers (chaetiger 303); dorsal simple chaetae thick and clearly bidentate (Fig. 2F), the ventral one very thin and slightly bidentate (Fig. 2G). Pharynx extending through 6 segments, anteriorly surrounded by 10 soft papillae, approximately 1.4 times longer than the length of proventricle, with a thin and acute mid-dorsal tooth inserted anteriorly. Proventricle extending through three segments, with 18 muscle cell rows (Fig. 2A). Three anal cirri, two lateral articulated with 13 articles and one mid-ventral smooth, very small (Fig. 5B–C).

#### Diagnosis

Palps free at their bases. Peristomium as long as first chaetiger. Dorsal cirri from mid-body spherical to bulbous with a long distal end, alternate with long and articulated cirri in an irregular pattern (they are not alternating one by one, both kinds of dorsal cirri may appear on the same segment). Bulbous and spherical dorsal cirri mostly with an unarticulated knob, but those from posterior segments can have two to four distal articles. Bidentate falcigers with short, subtriangular blade, proximal tooth slightly larger than distal one.

#### Etymology

The specific name refers to the irregular presence of bulbous and moniliform dorsal cirri.

#### Distribution

Only known from the type locality.

#### Ecology

The specimens were captured among biofoulers such as coralline algae, bryozoans, hydrozoans and tubes of polychaetes *Spirobranchus* spp., attached to pyramidal cement structures commonly used to fix coral *Pocillopora* spp. fragments.

#### Biology

Schizogamy. Formation of unique dicerous stolon.

#### Taxon discussion

*Parasphaerosyllisirregulata* sp. nov., differs from other species in the genus by having palps free at their base (*P.ezoensis*, *P.indica*, *P.malimalii* and *P.uschakovi* have palps fused basally) and the presence of an irregular alternation of bulbous and articulated dorsal cirri as follows: spherical to bulbous cirri with 1–3 terminal articulated knobs and moniliform ones, both distributed irregularly, sometimes a spherical/bulbous and moniliform cirrus may appear together within the same segment or only a spherical/bulbous cirrus may appear in several consecutive segments (not alternating one by one as occurs in the other species of the genus). Besides, its is important to emphasise the lost of bilateral symmetry of dorsal cirri in some segments: shape of dorsal cirrus from the right side of a particular segment is not always replicated in the left side as occurs in other species. Furthermore, the size and shape of falciger blades (short and subtriangular) with subequal teeth (proximal tooth slightly larger than the terminal one) is a unique feature (all other four species have falcigers with longer blades than in *P.irregulata* sp. nov. and proximal tooth being shorter than the terminal one).

The spherical or bulbous dorsal cirri of *P.irregulata* sp. nov. presumably have glands over its dorsal surface, aligned in several straight rows (Fig. 4E–F), some empty pores are visible, whereas other pores show short filaments or cilia arising from the holes. Their function is unknown, as well as their presence in *P.ezoensis and P.uschakovi*, but in the record of San Martín et al. (2008): figs. 19E–F, SEM, as "*P.indica*” from the Tasman Sea (specimen AM W30153), some pores are visible on the surface of bulbous dorsal cirri, but in the figure, it cannot be seen if the pores are aligned in rows as in *P.irregulata* or in a scattered pattern. Besides, the specimens from Tasman Sea and Western Australia reported by San Martín et al. (2008): fig. 22A, have bulbous dorsal cirri with dark, fibrillar inclusions forming nearly diagonal lines, as well as those illustrated by Capa et al. (2001): fig. 1D to *P.malimalli* and those here described in the holotype of *P.indica*. It is unknown if that pattern is distinctive of these three taxa or not or if these are related to pores or glands.

### Parasphaerosyllis malimalii

Capa, San Martín & López, 2001

B1F33AB8-E8AA-52B1-8A46-AAC65AF639ED

*Parasphaerosyllismalimalii* Capa, San Martín and López, 2001: 281, figs. 1–2.

#### Materials

- **Type status:** Other material. **Occurrence:** catalogNumber: UANL 8168; individualCount: 1; occurrenceID: 8226B633-08F1-5B4D-8766-7C761B753C2A; **Taxon:** phylum: Annelida; class: Polychaeta; order: Phyllodocida; family: Syllidae; genus: Parasphaerosyllis; specificEpithet: *malimalii*; **Location:** higherGeographyID: Tropical Eastern Pacific; higherGeography: Western Mexico; continent: America; waterBody: Pacific; island: Los Arcos; country: Mexico; countryCode: MX; stateProvince: Jalisco; municipality: Mismaloya; locality: Los Arcos Island; verbatimDepth: 2 m; decimalLatitude: 20.546583; decimalLongitude: -105.286694; **Identification:** identifiedBy: Gerardo Góngora-Garza, Jesús Angel de León-González; **Event:** eventDate: 26-08-2004; year: 2004; month: 08; day: 26; **Record Level:** institutionID: UANL, NL-INV-002-05-09; collectionID: UANL; institutionCode: UANL; collectionCode: UANL 8168; ownerInstitutionCode: Universidad Autónoma de Nuevo León
- **Type status:** Other material. **Occurrence:** catalogNumber: UANL 8169; individualCount: 1; occurrenceID: BF3D0549-FB5F-5C60-A0B5-553961D8761E; **Taxon:** phylum: Annelida; class: Polychaeta; order: Phyllodocida; family: Syllidae; genus: Parasphaerosyllis; specificEpithet: *malimalii*; **Location:** higherGeographyID: Tropical Eastern Pacific; higherGeography: Western Mexico; continent: America; waterBody: Pacific; country: Mexico; countryCode: MX; stateProvince: Nayarit; locality: Fideritas beach; verbatimDepth: intertidal; decimalLatitude: 21.026917; decimalLongitude: -105.295778; **Identification:** identifiedBy: Gerardo Góngora-Garza, Jesús Angel de León-González; **Event:** eventDate: 26-08-2004; year: 2004; month: 08; day: 26; **Record Level:** institutionID: UANL, NL-INV-002-05-09; collectionID: UANL; institutionCode: UANL; collectionCode: UANL 8169; ownerInstitutionCode: Universidad Autónoma de Nuevo León
- **Type status:** Other material. **Occurrence:** catalogNumber: UANL 8170; individualCount: 1; occurrenceID: BEE32DC3-B985-5DBD-867F-097B6AB61AF3; **Taxon:** phylum: Annelida; class: Polychaeta; order: Phyllodocida; family: Syllidae; genus: Parasphaerosyllis; specificEpithet: *malimalii*; **Location:** higherGeographyID: Tropical Eastern Pacific; higherGeography: Western Mexico; continent: America; waterBody: Pacific; country: Mexico; countryCode: MX; stateProvince: Nayarit; locality: Bajo del Toro; verbatimDepth: 1.5 m; decimalLatitude: 21.052778; decimalLongitude: -105.300889; **Identification:** identifiedBy: Gerardo Góngora-Garza, Jesús Angel de León-González; **Event:** eventDate: 26-08-2004; year: 2004; month: 08; day: 26; **Record Level:** institutionID: UANL, NL-INV-002-05-09; collectionID: UANL; institutionCode: UANL; collectionCode: UANL 8170; ownerInstitutionCode: Universidad Autónoma de Nuevo León

#### Description

The three specimens studied are incomplete posteriorly, the largest being 9.2 mm long and 0.60 mm wide. Body subcylindrical, flattened ventrally, pale yellowish, without pigmentation pattern, with 82 chaetigers. Prostomium oval, wider than long. Four eyes in a trapezoidal arrangement, the anterior pair larger than posterior one. Three articulated antennae, the middle one with approximately 50 articles, inserted in the posterior part of the prostomium between the basal pair of small eyes. Lateral antennae with 21 articles, inserted very close to the anterior edge of the prostomium. Palps short, sub-triangular, directed slightly towards ventral side, equal to or slightly smaller than the prostomium length, fused basally. No nuchal organs are observed. Peristomium slightly shorter than the first chaetiger, covering a small area of the prostomium. Two pairs of articulated tentacular cirri, the dorsal ones with some 40 articles and the ventral ones with 20. Dorsal cirri of the first chaetiger with 45–50 articles, chaetiger 2 (20–21 articles), chaetiger 3 (28–33 articles), chaetiger 4 (30–31 articles), chaetiger 5 (20–21 articles) and chaetiger 6 (37–39 articles). Alternating long and short anterior dorsal cirri with 38–40 and 23–25 articles, respectively. First bulbous cirrus appears at chaetiger 29, regularly alternating one by one with moniliform cirri (Fig. 6A). Elongated bulbous cirri with a smooth, non-articulated terminal knob. In the smallest specimen, the first bulbous dorsal cirrus appears on chaetiger 21. Moniliform dorsal cirri of the median region of the body with 20–22 articles. Parapodial lobe sub-conical, truncated, with two ligules, one anterior and one posterior. Ventral cirrus sub-triangular, inserted at the base, approximately half the length of the parapodial lobe. Anterior parapodia with 9–10 bidentate falcigers per bundle (Fig. 6B). Falcigers with dorso-ventral gradation approximately 2:1. With 5–6 chaetae per fascicle on middle parapodia. One or two pseudosimple chaetae formed by the thickening of the handle and loss of the blade in the dorsal position. Four to five bidentate falcigers with shorter and thicker blades than those of anterior parapodia (Fig. 6C). Posteriorly, the number of pseudosimple chaetae increases (3–4) (Fig. 6D) and that of falcigers decreases (1–2). With three aciculae on anterior segments, one of them with a straight tip and the other two with a slightly bent tip. Mid-body parapodia with two aciculae, both with the tip slightly bent and remaining so until the end of the incomplete specimen. Pharynx through 7–8 segments, with anterior medio-dorsal tooth. Approximately the same length as the proventricle, the latter being 32 muscle cell rings.

#### Diagnosis

Palps fused basally. Peristomium slightly shorter than the first chaetiger, covering a small area of the prostomium. Dorsal cirri from mid-body bulbous with a long distal end, alternating with long and articulate cirri in an irregular pattern (alternated one by one). Falcigerous bidentate with the secondary tooth very small, with pseudosimple setae formed by the thickening of the handle and the loss of the joint in the middle and posterior chaetigers. Pharynx and proventricle nearly the same size.

#### Distribution

Eastern Tropical Pacific, from Nayarit and Jalisco (Mexico) to Panama.

#### Ecology

Intertidal, associated with mats of coralline algae fixed to rocks at 2 m depth.

#### Biology

One of the specimens has a female dicerous stolon in poor condition of which the characteristic features cannot be seen.

#### Taxon discussion

This constitutes the first record of *Parasphaerosyllismalimalii* since its establishment. It is now reported from Nayarit and Jalisco (Central Mexican Pacific). *Parasphaerosyllismalimalii* is the only species within the genus to have pseudosimple chaetae formed by the thickening of the handle and loss of the blade. The specimens studied here have some differences from those originally reported to the Pacific of Panama. The median antenna is larger than original description (approx. 50 articles), the alternating anterior dorsal cirri are also larger than those reported by Capa et al. (2001) (with 38–40 and short with 23–25 articles) and the number of chaetae per bundle in anterior chaetigers is also larger (9–10) than the holotype. These differences are probably due to ontogeny.

## Identification Keys

### Taxonomic key to species of *Parasphaerosyllis* Monro, 1937za

**Table:** 

1	Pseudosimple chaetae present (formed by the loss of the blade and thickening of the shaft)	*P.malimalii* Capa, San Martín & López, 2001
–	Pseudosimple chaetae absent	2
2	Moniliform dorsal cirri alternating one by one with bulbous cirri; falcigerous with normal shape	3
–	Moniliiform dorsal cirri irregularly alternating with bulbous cirri, not one by one; falcigers with small, sub-triangular blade	*P.irregulata* sp. nov.
3	Median antenna inserted into the posterior part of the prostomium, between the posterior eyes	4
–	Median antenna inserted in the middle part of the prostomium, between the anterior eyes	*P.ezoensis* Imajima & Hartman, 1964
4	Falcigerous bidentate with smooth blade on its inner edge (without small teeth or tertiary teeth)	*P.uschakovi* Chlebovitsch, 1959
–	Falcigerous bidentate with serrated blade on its inner edge (with small teeth or tertiary teeth)	*P.indica* Monro, 1937

## Discussion

This note deals with the re-description of a widely-reported syllid genus and the recognition of a new species from Mexico. It reveals the necessity of a revision of all worldwide records of *P.indica* taking into account the informative features described in this contribution for the recognition of species in *Parasphaerosyllis*. In addition, molecular studies of *P.indica* are recommended. Apparently, there is a barcode available as indicated at the web page of the British Museum of Natural History, but it is unknown if it belongs to the holotype or to another specimen (British Museum of Natural History 2023). No sequences are available in BOLD SYSTEMS or NCBI depositories.

## Supplementary Material

XML Treatment for Parasphaerosyllis indica

XML Treatment for Parasphaerosyllis irregulata

XML Treatment for Parasphaerosyllis malimalii

## Figures and Tables

**Table 1.** Reports of *Parasphaerosyllisindica* Monro, 1937 around the globe presented in chronological order. Only records that include diagnosis, drawings or some information were here considered.

Report	Locality	Features
Fauvel 1939: 298	French Indochina (Cauda Reef, Annam, now Vietnam)	Some specimens with a male stolon, with proventricle as long as 3 segments (in segments 7–9, 9–11 or 12–14, respectively).
Fauvel 1950: 351	Dakar, Senegal	Bulbous dorsal cirri appears on chaetiger 15.
Fauvel 1953: 162, figs. 80c–d (figures were re-drawn from Monro (1937))	Arabian coast	Palps fused at the base; proventricle short; bulbous cirri appears on chaetiger 16, with a small terminal knob; alternation of dorsal cirri one by one.
Rioja 1958: 246-251, figs. 21–27	Isla Verde, Gulf of Mexico	Peristomium as long as subsequent segments; proventricle as long as 3–4 segments; bulbous dorsal cirri appear on chaetiger 26; bulbous cirri with short to long cirrophores; bulbous cirri with one terminal article; simple chaetae present.
Fauvel and Rullier 1959: 514–515	Gorea Island, Senegal	Bulbous dorsal cirri appears in chaetiger 5; three pairs of moniliform cirri alternating with bulbous cirri.
Rullier 1964: 165	Cameroon	Median antena inserted posterior to the inferior eyes; lateral antennae at the same level of eyes; dorsal cirri from anterior segments with 30–35 articles; bulbous dorsal cirri appearing in chaetiger 23, and “filled with a black substance”
Hartmann-Schröder 1965: 115	Maui, Hawaii	Specimen with 34 chaetigers; stolon with 12 segments.
Rullier 1972: 69	New Caledonia	“Fits perfectly with Monro's description (1937)”. First 15 dorsal cirri being moniliform; alternation of moliniform and bulbuls cirri one by one in posterior segments.
Westheide 1974: 64-67, figs. 27–29	Galapagos	Median antenna with up of 54 articles; lateral antennae with 23–24 articles; dorsal tentacular cirri with 43 articles, ventral with 22 articles; first pair of dorsal cirri with 60 articles; bulbous dorsal cirri starting at chaetiger 26, with a terminal knob; alternation one by one; proventricle with 20 rows of muscle cells.
Cantone 1976: 230	Somalia	Proventricle as long as 5 segments; bulbous cirri appear at chaetiger 16; bulbous cirri with terminal articles.
Hartmann-Schröder 1980: 49	Exmouth, tropical northwest Australia	Specimen with 44 segments; bulbous dorsal cirri staring at chaetiger 14, terminal knobs with 2 articles; proventricle with 22–25 muscle cells.
Hartmann-Schröder 1987: 32	Point Lonsdale, Victoria, Australia	Specimen with 55 segments; bottle-shaped cirri (bulbous) alternating with articulated cirri; bulbous cirri with knobs and 2 terminal articles; without simple chaetae; proventricle with 22 muscle cells.
Hartmann-Schröder 1991: 27	Heron Island, Queensland, Australia	Dorsal cirri with 15–17 articles; bulbous dorsal cirri appearing in chaetiger 14; proventricle as long as 5.5 segments.
San Martín et al. 2008: 146, figs. 19e–f, 22a–e, 24a–b	Tasman Sea, Australia	Peristomium shorter than subsequent segments; median antenna with up to 54 articles; lateral antenna with up to 24 articles; dorsal tentacular cirrus with up to 43 articles; ventral tentacular cirri with up to 22 articles; “lemon-like”bulbous dorsal cirri from the proventricle to the end of the body, with distinct cirrophore; proventricle extending 7–8 segments; dorsal cirri alternating one by one; bulbous cirri with diagonal black lines; simple chaetae present.
Liñero-Arana and Díaz-Díaz 2011: 24, figs. 4.8–4.10	Venezuela	Dorsal tentacular cirri with 32–51 articles; ventral tentacular cirri with 18–23 articles; ovoid dorsal cirrus appears on chaetiger 24, with small distal button (knob); falcigers bidentate, with sub distal teeth small; simple chaetae present; proventricle as long as 3–5 chaetigers with 25–28 rows of muscle cells.
Aguado et al. 2015: 49, fig. 5F	Lizard Island, Australia	“Antennae longer than those described by San Martín et al. (2008)”, it could be interpreted as having more than 24 articles; anal papilla inflated, similar to spherical dorsal cirri (it suggesting that dorsal cirri are spherical).
Cañete 2017: 1070-1071, figs. 1a–g	Easter Island, Chile	Palps fused at the base; peristomium shorter than subsequent segments; proventricle extend 7–8 segments with 22 to 27 rows of muscle cells; bulbous dorsal cirrus appears in the chaetiger 19; bulbous dorsal cirri with diagonal lines; simple chaetae present.
